# Secretion of Recombinant Interleukin-22 by Engineered Lactobacillus reuteri Reduces Fatty Liver Disease in a Mouse Model of Diet-Induced Obesity

**DOI:** 10.1128/mSphere.00183-20

**Published:** 2020-06-24

**Authors:** Jee-Hwan Oh, Kathryn L. Schueler, Donnie S. Stapleton, Laura M. Alexander, Chi-Liang Eric Yen, Mark P. Keller, Alan D. Attie, Jan-Peter van Pijkeren

**Affiliations:** a Department of Food Science, University of Wisconsin—Madison, Madison, Wisconsin, USA; b Department of Biochemistry, University of Wisconsin—Madison, Madison, Wisconsin, USA; c Department of Nutritional Sciences, University of Wisconsin—Madison, Madison, Wisconsin, USA; University of California, Davis

**Keywords:** diet-induced metabolic syndrome, fatty liver disease, IL-22, *Lactobacillus reuteri*, probiotic, engineered probiotic, interleukin-22, nonalcoholic fatty liver disease, steatosis

## Abstract

The incidence of metabolic syndrome continues to rise globally. In mice, intravenous administration of interleukin-22 (IL-22) ameliorates various disease phenotypes associated with diet-induced metabolic syndrome. In patients, oral treatment is favored over intravenous treatment, but methodologies to deliver IL-22 via the oral route are nonexistent. The goal of this study was to assess to what extent engineered Lactobacillus reuteri secreting IL-22 could ameliorate nonalcoholic fatty liver disease. We used a mouse model of diet-induced obesity and assessed various markers of metabolic syndrome following treatment with L. reuteri and a recombinant derivative. Mice that received an 8-week treatment of wild-type probiotic gained less weight and had a smaller fat pad than the control group, but these phenotypes were not further enhanced by recombinant L. reuteri. However, L. reuteri secreting IL-22 significantly reduced liver weight and triglycerides at levels that exceeded those of the probiotic wild-type treatment group. Our findings are interesting in light of the observed phenotypes associated with reduced nonalcoholic liver disease, in humans the most prevalent chronic liver disease, following treatment of a next-generation probiotic that is administered orally. Once biological and environmental containment strategies are in place, therapeutic applications of recombinant Lactobacillus reuteri are on the horizon.

**IMPORTANCE** In humans, nonalcoholic fatty liver disease (NAFLD) is the most prevalent liver disease due to the increased prevalence of obesity. While treatment of NAFLD is often geared toward lifestyle changes, such as diet and exercise, the use of dietary supplements such as probiotics is underinvestigated. Here, we report that probiotic Lactobacillus reuteri reduces fatty liver in a mouse model of diet-induced obesity. This phenotype was further enhanced upon delivery of recombinant interleukin-22 by engineered Lactobacillus reuteri. These observations pave the road to a better understanding of probiotic mechanisms driving the reduction of diet-induced steatosis and to development of next-generation probiotics for use in the clinic. Ultimately, these studies may lead to rational selection of (engineered) probiotics to ameliorate fatty liver disease.

## Abstract (precis)

In humans, nonalcoholic fatty liver disease (NAFLD) is the most prevalent liver disease due to the increased prevalence of obesity. While treatment of NAFLD is often geared toward lifestyle changes, such as diet and exercise, the use of dietary supplements such as probiotics is underinvestigated. Here, we report that probiotic Lactobacillus reuteri reduces fatty liver in a mouse model of diet-induced obesity. This phenotype was further enhanced upon delivery of recombinant interleukin-22 by engineered Lactobacillus reuteri. These observations pave the road to a better understanding of probiotic mechanisms driving the reduction of diet-induced steatosis and to development of next-generation probiotics for use in the clinic. Ultimately, these studies may lead to rational selection of (engineered) probiotics to ameliorate fatty liver disease.

## INTRODUCTION

The global epidemic of metabolic syndrome is a pressing health concern. Individuals diagnosed with metabolic syndrome are more susceptible to cardiovascular disease, type 2 diabetes, fatty liver, and some cancers, including breast, pancreatic, colorectal, and prostate cancers (1). Key circulatory diagnostic markers of metabolic syndrome are hyperglycemia, high-density lipoprotein, and triglyceride (TG) levels, and one of the key risk factors of metabolic syndrome is obesity (body mass index [BMI] of ≥30 kg/m^2^) (1). In obese individuals, the liver is a key organ presenting abnormalities in glucose and lipid metabolism, which are associated with increased incidence of nonalcoholic fatty liver disease. As of today, behavioral changes are considered the main driver to revert metabolic syndrome, but medical intervention strategies are considered the first line of treatment (2). In both nonalcoholic and alcoholic liver disease models, interleukin-20 (IL-20) family cytokines reduce liver injury and inflammation (3). For example, interleukin-22 (IL-22), a member of the IL-20 subfamily, controls lipid metabolism in the liver via activation of the STAT3 signaling pathway and, consequently, reduces fatty liver disease (4). Therefore, IL-22 has great potential to serve as a therapeutic protein to reduce alcohol- and non-alcohol-induced fatty liver diseases (5).

Currently, eukaryotic and prokaryotic cells are exploited as factories to produce therapeutic peptides and proteins (TPPs). Due to instability and large complex structure, most TPPs are administered parenterally by intramuscular, intravenous, or subcutaneous injection. But there are disadvantages of injection, which leads to cannula-related infections and adverse reactions such as pain, discomfort, and skin necrosis (6). Therefore, nonparenteral routes such as oral, nasal, transdermal, and rectal administration have been tested to deliver TPPs. Among these alternatives, oral administration is the most favored route in patients (7).

**FIG 1:**
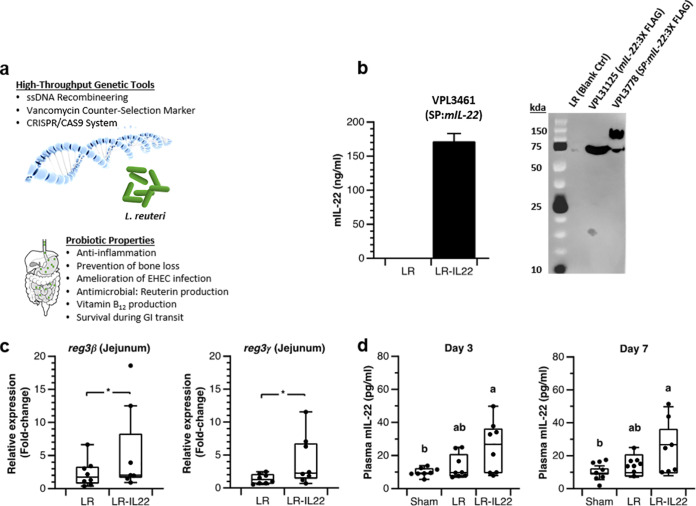
Probiotic properties of L. reuteri 6475 and systemic increase of biologically active mIL-22 by engineered L. reuteri. (a) Genetic tool availability (top) and beneficial probiotic properties (bottom). (b) (Left) mIL-22 ELISA on bacterial supernatant of wild-type L. reuteri and L. reuteri VPL3461 expressing mIL-22; (right) Western blot analysis of trichloroacetic acid (TCA)-precipitated proteins derived from L. reuteri supernatant (control [Ctrl]) and total lysates of L. reuteri strains VPL31125 and VPL3778 for mIL-22 production in LDM3 at 37°C and an OD_600_ of 3.5. SP, signal peptide. (c and d) Eight-week-old male C57BL/6 mice (*n* = 8 per group) were administered nothing (sham), L. reuteri (10^9^ cells/mouse), and L. reuteri expressing IL-22 (10^9^ cells/mouse). (c) Fold change in *reg3β* (left) and *reg3γ* (right) gene expression in the jejunum after the consecutive oral administration of L. reuteri and L. reuteri expressing IL-22 for 7 days. Numbers indicate fold change relative to the mean value of the sham group. (d) Mice were gavaged daily for 7 days. Plasma mIL-22 levels were determined by ELISA. One-way ANOVA with Tukey’s HSD test was used to determine the levels within three groups (a, b, or ab). The data in panels b to d represent averages of three biological replicates. For the box plots (c and d), center lines show the medians; box limits indicate the 25th and 75th percentiles. Error bars represent standard deviations. ns, no statistical significance. *, *P < *0.05; **, *P < *0.01 (*t* test).

Since the 1990s, live bacteria have been developed as factories for production of recombinant human proteins, including interleukin-1β, -2, and -6 (8, 9). Especially, food-grade bacteria engineered to produce immunomodulatory molecules offer exciting potential to reduce chronic diseases. For example, the cheese starter lactic acid bacterium Lactococcus lactis MG1363 was engineered to secrete IL-10. In a mouse model of colitis, oral administration of recombinant L. lactis reduced intestinal inflammation, and the results of phase I clinical trial demonstrated safety (10). This seminal work laid the foundation to develop lactic acid bacteria as *in situ* TPP delivery vectors. The organism pertinent to this study, Lactobacillus reuteri, has proven to be functional as a therapeutic delivery platform in animal disease models (11). Advantages of using L. reuteri are the availability of high-throughput genome editing tools (12, 13) and its ability to thrive in the gut ecosystem, and L. reuteri has—compared to other lactobacilli and L. lactis—an extraordinarily low mutation rate (14), which is expected to improve the genetic stability of the recombinant organism. Lastly, L. reuteri ATCC PTA 6475, the strain we used in this study, has probiotic features, including amelioration of obesity (15) (Fig. 1a).

The goal of this study was to investigate to what extent engineered L. reuteri can further boost the native ability to ameliorate markers of diet-induced obesity. We demonstrated that the recombinant IL-22 delivered by L. reuteri is biologically active and that—compared to that of wild-type L. reuteri—the therapeutic efficacy was enhanced, specifically in the reduction of liver weight and triglycerides. This work, therefore, laid the foundation to develop L. reuteri as a next-generation probiotic to combat fatty liver disease.

## RESULTS

### Construction of a recombinant L. reuteri strain that secretes murine IL-22.

We cloned a fusion of a Lactobacillus reuteri signal peptide and the coding sequence of murine interleukin-22 (mIL-22) in the high-copy number plasmid pJP028 (Table S1), which was established in L. reuteri to yield L. reuteri expressing IL-22. By ELISA, we demonstrated that L. reuteri expressing IL-22 produced up to 171 ng/ml of mIL-22 in LDM3 medium (Fig. 1b). We did not use MRS medium, as this interfered with enzyme-linked immunosorbent assay (ELISA) (data not shown). Also, Western blot analysis of a C-terminally FLAG-tagged version of mIL-22 without signal peptide revealed that mIL-22 migrates as a tetramer (Fig. 1b), which is in line with an observation made previously (16). When we analyzed the supernatant of recombinant L. reuteri that is predicted to secrete mIL-22, we observed that only 18.3% of the recombinant protein was cleaved and migrated as a tetramer, whereas 81.7% recombinant protein appeared to be a fusion of the signal peptide and mIL-22, suggesting suboptimal processing of the signal peptide (Fig. 1b). The total amount of recombinant protein detected in the supernatant represented mature and unprocessed mIL-22 (data not shown).

10.1128/mSphere.00183-20.2 TABLE S1 Bacterial strains and plasmids used in this study. VPL, Van Pijkeren Laboratory strain identification number; pVPL, Van Pijkeren Lab plasmid identification number. Download Table S1, DOCX file, 0.01 MB. Copyright © 2020 Oh et al. 2020 Oh et al. This content is distributed under the terms of the Creative Commons Attribution 4.0 International license.

### L. reuteri produces biologically active mIL-22.

Since part of the recombinant protein appears to be dissociated from its signal sequence, i.e., mature, we tested next if mIL-22 delivered by L. reuteri was biologically active in the mouse intestinal tract. For 7 consecutive days, mice were gavaged daily with 10^9^ cells of L. reuteri or L. reuteri expressing IL-22. Jejunum samples were harvested, followed by transcriptional analyses of *reg3β* and *reg3γ*, genes that are both regulated by IL-22 (4). Relative to the mice administered L. reuteri (LR group mice), mice administered L. reuteri expressing IL-22 (LR-IL-22 group mice) showed increased gene expression of *reg3β* (2.6-fold [*P = *0.03]) and *reg3γ* (2.9-fold [*P = *0.04]) (Fig. 1c). In the same mouse experiment, 1 week of treatment with L. reuteri expressing IL-22 yielded a subtle but statistically significant increase in plasma mIL-22 levels compared to those in the sham group, but there was no difference from the LR group (Fig. 1d). While this is an interesting observation, at this point, it remains unclear what the underlying mechanism of the systemic increase of IL-22 following oral administration of L. reuteri VPL3461 is, and this warrants further investigation.

### Recombinant L. reuteri reduces fatty liver in a mouse model of diet-induced obesity.

**FIG 2:**
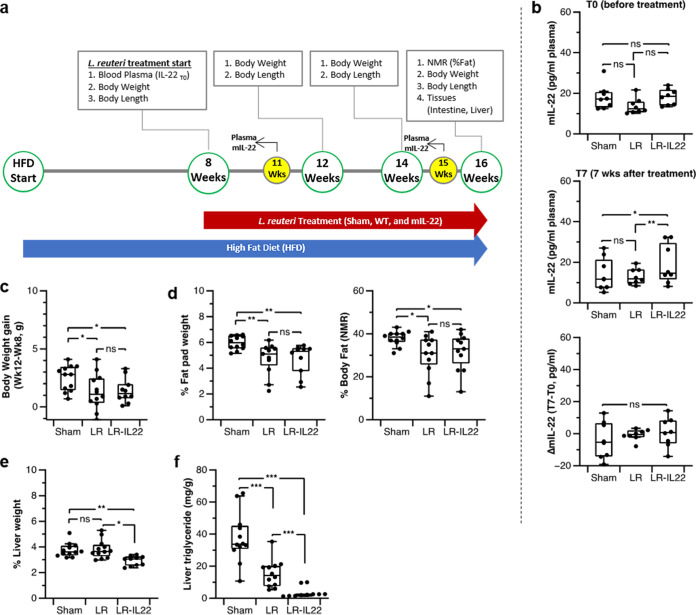
L. reuteri VPL3461 (LR-IL-22 group) improves diet-induced fatty liver disease. (a) Outline of high-fat-diet-induced obesity (DIO) *in vivo* model. Twelve mice per group were used for the sham, LR (wild-type), and LR-IL-22 (mIL-22) groups. Mice in the LR and LR-IL-22 groups were orally administered 10^9^ CFU/mouse/day. (b) Plasma mIL-22 quantification. Blood plasma was isolated from the mice before and after the oral treatment. Plasma mIL-22 levels were determined by ELISA. (c) Mouse body weight monitored during treatment. Weight gain represents body weight difference between weeks 12 and 8 (T0). (d) Abdominal percent fat pad and total percent body fat at 16 weeks. (e) Percent liver weight. Whole liver weights were compared for the three groups at 16 weeks. For panels d and e, fat compositions and liver weight were calculated per body weight. (f) One-hundred-milligram left top liver lobe samples were collected from the whole liver, followed by quantification of liver triglyceride. For panels b to f, center lines show the medians; box limits indicate the 25th and 75th percentiles. Error bars represent standard deviation. *, *P < *0.05; **, *P < *0.01; ***, *P < *0.001 (*t* test).

Previously, we demonstrated that recombinant L. reuteri—which released mIL-22 following bacteriophage-mediated lysis—ameliorates steatohepatitis in a mouse model of alcohol-induced liver disease (11). To determine to what extent L. reuteri, and its recombinant derivative secreting mIL-22, impacts markers of metabolic syndrome, including fatty liver, we used a mouse model of diet-induced obesity. For 16 weeks, animals received a high-fat, high-sucrose diet (45% fat and 41% carbohydrate kcal). Starting at week 8 until the end of the trial (week 16), animals were subjected to daily treatment with LDM3 medium only (sham), L. reuteri, or L. reuteri expressing IL-22 (Fig. 2a). Following 7 weeks of treatment with L. reuteri expressing IL-22, mIL-22 plasma levels were significantly raised compared to those in the LR and the untreated control groups (Fig. 2b). However, before treatment (T0) with bacteria, the group to receive L. reuteri expressing IL-22 had elevated mIL-22 levels compared to those of the other treatment groups, yet differences were not statistically significant (*P > *0.05). When we tracked the differences in plasma mIL-22 in each mouse over the 7-week period (T7 to T0), values were not found to be statistically different. Thus, while the data trended toward increased plasma mIL-22 levels following treatment with L. reuteri expressing IL-22, these data need to interpreted with care given the increased basal levels of plasma mIL-22 in the LR-IL-22 group prior to the start of the treatment.

In terms of body weight gain and body fat composition, mice treated with L. reuteri or L. reuteri expressing IL-22 gained less weight (−47.7% LR versus sham [*P = *0.04] and −39.2% LR-IL-22 versus sham [*P = *0.03]) and had a lower body fat composition (−21.3% LR [*P = *0.004] and −19.0% LR-IL-22 [*P = *0.003]) and percent fat pad weight (−20.6% LR [*P = *0.01] and −15.6% LR-IL-22 [*P = *0.03]) than mice in the sham treatment group. The reduction in percent fat pad weight was representative of percent body fat, as determined by the body composition (percent fat mass) using nuclear magnetic resonance (NMR) (−7.8% LR [*P = *0.02] and −6.4% LR-IL-22 [*P = *0.04]). However, no statistical differences were observed when comparing these markers between the LR and LR-IL-22 groups (Fig. 2c and d).

The largest impact of treatment with recombinant probiotic was found in markers related to liver disease. The LR-IL-22 group displayed reduced percent liver weight compared to those of the LR group (−22.3% [*P = *0.002]) and the sham group (−20.7% [*P < *0.001]), while percent liver weight between the LR and the sham groups was not different (*P > *0.05) (Fig. 2e). Liver triglyceride levels were also reduced in animals in the LR-IL-22 group compared to those in the LR treatment group (−4.6-fold [*P < *0.001]) and the sham group (−11.4-fold [*P < *0.001]). We determined that L. reuteri treatment also reduced liver triglyceride levels compared to those in the sham group (−2.5-fold [*P < *0.001]) (Fig. 2f). Conclusively, in a model of diet-induced metabolic syndrome, wild-type L. reuteri inhibited weight gain and body fat accumulation and reduced liver fat and liver triglycerides; liver fat and triglycerides were further reduced following the delivery of L. reuteri expressing IL-22. Thus, these data emphasize the potential to explore a recombinant probiotic to combat nonalcoholic fatty liver disease, the most prevalent chronic liver disease worldwide (17).

## DISCUSSION

In this study, we demonstrated the proof of concept that engineered L. reuteri can secrete biologically active mIL-22, which reduced markers related to liver disease in a model of diet-induced obesity. We believe that our study, combined with recent studies demonstrating the efficacy of recombinant L. reuteri in animal disease models of total body irradiation and alcohol-induced liver disease (11, 18), reveals the potential to develop L. reuteri as a next-generation probiotic to treat disease in the clinic.

To export recombinant mIL-22, we relied on the native bacterial secretion machinery. As is evident from our Western blot analysis, only part of the recombinant protein was processed correctly by the bacterial cell secretion machinery. We made a similar observation with leptin (14). While incomplete cleavage may be overcome by testing different leader sequences or by optimizing the combination of the leader sequence and the N-terminal sequence of the mature protein, these are not trivial tasks (19). Alternative approaches may therefore be more efficient. For example, we recently demonstrated that activation of native prophages in L. reuteri can lyse the cell and subsequently release a recombinant mature protein that has accumulated inside the cell; this approach has proven successful in preclinical disease models (11, 18). Using bacterial lysis to release mature proteins also has the advantage that lysis can be optimized to contribute to containment. Future studies will investigate differences in the therapeutic efficacy between the two probiotic delivery systems in the diet-induced obesity model.

Regardless of the partial processing of mIL-22, we observed in mice fed a regular chow and administered L. reuteri expressing IL-22 a subtle but significant increase in systemic IL-22 levels (Fig. 1d), which was also observed in mice following 7 weeks of treatment with a high-fat diet (HFD) and L. reuteri expressing IL-22 (Fig. 2b). While future studies need to validate the biological relevance of differences in systemic IL-22 during HFD treatment, probiotic-driven delivery of mIL-22 clearly impacts host physiology. For example, after 8 weeks of treatment with an HFD combined with L. reuteri expressing IL-22, the total body length of mice—based on nose-tail measurements—was greater than that of animals that received L. reuteri or sham treatment (Fig. S1a), and our data show a weak correlation between body length and growth hormone level during treatment with L. reuteri expressing IL-22 (Fig. S1). El-Zayadi et al. recently showed that IL-22 has a postinflammation osteogenic property in the absence of gamma interferon (IFN-γ) and tumor necrosis factor (TNF) by regulating mesenchymal stem cells (MSC), which play a role in new bone formation and repair (20). The largest impact of probiotic-mediated delivery of IL-22 in our model of diet-induced obesity was reduction of liver triglycerides. Indeed, IL-22 has several metabolic benefits, as it decreases chronic inflammation and regulates lipid metabolism in liver and adipose tissues (4, 5). In mice, IL-22 elicits hepatoprotective properties via the activation of STAT3 (3, 5, 21); STAT3 downregulates the expression of lipogenic genes—and consequently reduces fat accumulation—and upregulates the expression of antiapoptotic genes, including *reg3*. Because the IL-22/STAT3/*reg3* axis exists in the intestine (21), it is plausible that IL-22 upregulates REG3β and REG3γ in jejunum via STAT3. Especially, REG3 is a multifunctional enzyme overexpressed by IL-22 induction and known to reduce injury in liver and intestine. Antiapoptotic activity of REG3β and REG3γ in liver and gut epithelium is essential in tissue regeneration via activation of protein kinase A, while antimicrobial activity of intestinal REG3 prevents translocation of bacteria to the liver (11, 21).

10.1128/mSphere.00183-20.1 FIG S1 Body length change and growth hormone level after treatment. (a) Body length changes were monitored before (7 weeks) and after (14 weeks) treatment. (b) Growth hormone in blood plasma was tested after 14 weeks on a high-fat diet (HFD). ns, no statistical significance. *, *P < *0.05; ***, *P < *0.001 (*t* test). Download FIG S1, TIF file, 0.1 MB. Copyright © 2020 Oh et al. 2020 Oh et al. This content is distributed under the terms of the Creative Commons Attribution 4.0 International license.

Treatment with L. reuteri expressing IL-22 yielded subtle but statistically significant changes in the expression of *reg3* genes in the small intestine and in IL-22 levels in the plasma. However, not all animals responded to treatment with L. reuteri expressing IL-22. At this point, the rationales for different responses among animals within the same treatment group are merely speculative. Perhaps differences in the microbiota between mice housed in different cages could affect L. reuteri physiology and IL-22 production. Also, additional insight in the production of IL-22 throughout the gastrointestinal tract, along with measuring *reg* expression and plasma IL-22 levels in diseased mice, will be helpful to better understand the observed inconsistencies between animals. For future studies—if direct comparisons were to be made between different animal trials—it is expected that animals representing the control groups would be treated identically, though this was not a concern in the current study. The use of REG3 mutant mice, as previously described in a model of ethanol-induced liver disease (11), could provide more mechanistic insight how recombinant IL-22 reduces fatty liver disease in our model. As of now, the lack of such studies represents a limitation of our work; nevertheless, our observations provide a clear foundation for the above-mentioned future studies to gain a deeper mechanistic understanding of how L. reuteri-mediated delivery of IL-22 ameliorates fatty liver disease. Finally, while recombinant (probiotic) bacteria hold the potential to treat and prevent disease, the development and assessment of environmental and biological containment systems are critically important to bring these engineered bacteria a step closer to the clinic.

## MATERIALS AND METHODS

### Bacterial strains, plasmids, and media.

All bacterial strains and plasmids used in this study are listed in Table S1. Lactobacillus reuteri 6475 and its derivatives were cultured at 37°C in deMan Rogosa Sharpe (MRS) medium (Difco, BD Biosciences). Where appropriate, erythromycin was added to a final concentration of 5 μg/ml for L. reuteri. Electrocompetent cells of L. reuteri were prepared as described before (22). To test mIL-22 expression and to prepare bacteria for animal experiments, L. reuteri was cultured in lactobacillus defined medium III (LDM3) (23).

### Reagents and enzymes.

Cloning was performed via ligase cycling reaction (LCR) (24). Enzymes and reagents for LCR were purchased from Lucigen. PCR for cloning purposes was performed with the high-fidelity enzyme Phusion Hot Start polymerase II (Fermentas). PCR for screening purposes was performed with *Taq* polymerase (Denville Scientific). To concentrate the LCR before electrotransformation into L. reuteri, we used Pellet Paint coprecipitant (Novagen). Oligonucleotides and synthetic double-stranded DNA fragments were purchased from Integrated DNA Technologies (IDT). All oligonucleotides and synthetic DNA fragments used in this study are listed in Table S2.

10.1128/mSphere.00183-20.3 TABLE S2 Oligonucleotides and synthetic DNA used in this study. oVPL, oligonucleotide identification number; gVPL, synthetic DNA identification number. Download Table S2, DOCX file, 0.01 MB. Copyright © 2020 Oh et al. 2020 Oh et al. This content is distributed under the terms of the Creative Commons Attribution 4.0 International license.

### Plasmid construction for mIL-22 secretion from L. reuteri 6475.

We engineered Lactobacillus reuteri 6475 to secrete the murine cytokine interleukin-22 (mIL-22). We opted for expression from the multicopy plasmid pJP028 (a gift from Robert Britton) to maximize mIL-22 production (Table S1). By PCR (oVPL1221 and oVPL1222) we amplified the backbone of pJP028, omitting the cell wall anchor domain, to yield a 4.579-kb product. For optimal expression of mIL-22 in our expression host L. reuteri, we first applied *in silico* codon optimization of the mIL-22 coding sequence using the online software OPTIMIZER (http://genomes.urv.es/OPTIMIZER/), followed by gBLOCK (IDT) synthesis. The synthetic product (gVPL1 [Table S2]) was amplified by PCR (oVPL1219 and oVPL1220), followed by fusion to the pJP028 backbone by LCR (24). The LCR mixture was precipitated and transformed L. reuteri 6475. Transformants were screened by PCR (oVPL329 and oVPL363) to confirm the cloning of *mIL-22*. One positive clone was colony purified, a 1.584-kb mIL-22 secretion cassette was confirmed by colony PCR, and the integrity of the construct was confirmed by DNA sequencing (GeneWiz). The resultant strain was named VPL3461.

To insert a C-terminal 3× FLAG tag in IL-22, we used oVPL2113 and oVPL2114, which contain 3× FLAG sequence tags, and performed inverse PCR of the plasmid backbone followed by self-circularization. The resultant plasmids were named pVPL3776 (SP:IL-22:3X FLAG) and pVPL31125 (IL-22:3X FLAG) (Table S1).

### Culture conditions for mIL-22 secretion.

L. reuteri 6475 stored at –80°C was inoculated into 10 ml of MRS broth, and L. reuteri VPL3461 was inoculated into 10 ml of MRS medium containing 5 μg/ml of erythromycin at 37°C. An overnight culture of each strain was subcultured into the same medium (10 ml), and initial optical density at 600 nm (OD_600_) was set at 0.1. Cells from each culture were harvested at mid-log phage (OD_600_ = 1.0 to ∼1.2) by centrifugation at 5,000 rpm for 5 min. Cells were washed twice in LDM3 containing glycerol (15%, vol/vol). Washed-cell pellet was kept at –80°C until use or resuspended in 10 ml of fresh prewarmed LDM3 and incubated at 37°C until reaching an OD_600_ of 3.5 to ∼3.7. Cells in LDM3 were 10-fold concentrated in 1 ml of LDM3 culture supernatant after centrifugation at 3,214 × *g* for 5 min. One hundred microliters of concentrated cell suspension in LDM3 was used for oral administration, and the supernatant was used to quantify mIL-22 level by ELISA (R&D Systems).

### mIL-22 analysis.

L. reuteri strains 6475 and VPL3461 were cultured in LDM3 as described under “Culture conditions for mIL-22 secretion,” and the supernatants were collected after centrifugation (5 min at 3,214 × *g*), followed by filter sterilization (0.22 μm; Millipore). One hundred microliters of filter-sterilized supernatant from L. reuteri 6475 and VPL3461 was assessed for the presence of mIL-22 by ELISA (R&D Systems).

For Western blot analysis, samples from L. reuteri strains engineered to secrete or intracellularly accumulate mIL-22 (VPL3778 or VPL31125, respectively) were prepared as previously described (14), with the exception that protein from VPL3778 was precipitated from a higher volume of culture (35 ml).

### mIL-22 treatment in a mouse model.

Twenty-four 6-week-old male B6 mice (C57BL/6J) were purchased from The Jackson Laboratory (Bar Harbor, ME). Animals were housed at an environmentally controlled facility with a 12-h light and dark cycle. Both diet (Formulab 5008; LabDiet, St. Louis, MO) and water were freely available to the animals. After transport, animals were allowed to adjust to the new environment for 2 weeks, after which treatment by oral gavage started. Three groups (*n* = 8 per group) were treated daily for 7 consecutive days. Treatments were a sham gavage in which the animals were subjected to insertion of a gavage needle without administering anything (sham group), L. reuteri 6475 (LR group), and L. reuteri VPL3461 (LR-IL-22 group). The bacterial load administered per mouse was ∼1 × 10^9^ CFU in a volume of 100 μl of the respective bacterial supernatant.

### A mouse model of high-fat-diet-induced metabolic syndrome.

Thirty-two 6-week-old male C57BL/6J mice were purchased from The Jackson Laboratory (Bar Harbor, ME). The animals were housed (*n* = 3 per cage) in an environmentally controlled facility with a 12-h light and 12-h dark cycle for 8 weeks on a high-fat diet (TD.08811; Envigo) before L. reuteri treatment. Food and water were provided *ad libitum* during the mouse experiment. After 8 weeks on the high-fat diet, mice (*n* = 12 per group) were gavaged daily for 2 consecutive months with 100 μl of LDM3 (sham group), a culture containing 10^9^ CFU/ml of L. reuteri 6475 (LR group), or a culture containing 10^9^ CFU/ml of L. reuteri VPL3461 (LR-IL-22 group). Retro-orbital bleeding was performed at 8, 11, and 15 weeks. Body weight and length were monitored every other week. At the endpoint (16 weeks), body composition was monitored via NMR analysis followed by CO_2_ euthanization for sampling tissues (Fig. 2a).

### Blood plasma isolation.

Two hours after the last gavage, 50 μl of blood per mouse was collected via retro-orbital puncture using heparin-coated capillary tubes (Fisher Scientific). Plasma was isolated from the whole-blood sample by centrifugation at 9,000 rpm for 7 min, and the plasma fraction was stored at –80°C until use. By ELISA (as described above), we determined plasma mIL-22 levels.

### NMR body composition analysis.

After the 16 weeks on the HFD, total body fat composition from the live animals was measured by using NMR (4-in-1-1000 analyzer; EchoMRI, Houston, TX). Percent fat composition was calculated based on the amount of body fat on total mass (fat plus lean).

### Liver triglyceride analysis.

Liver triglycerides (TGs) were quantified following the Jouihan method (25). One hundred milligrams of frozen liver tissue was homogenized, and total lipids were extracted in ethanolic KOH at 55°C for 16 h. Triglyceride content was determined by colorimetric analysis using free glycerol reagent (Sigma-Aldrich; F6428) and glycerol standard (Sigma-Aldrich; G7793).

### cDNA synthesis.

To assess the biological functionality of L. reuteri-secreted mIL-22, we assessed gene expression levels of *reg3β* and *reg3γ*. Part of the small intestine (jejunum) of each animal was processed for RNA isolation. First, samples were homogenized (Omni TH; Omni International), followed by RNA isolation and on-column DNase I treatment (Qiagen). Following isolation, additional DNase I treatment was conducted (RQI DNase; Promega), followed by quantification using a Qubit fluorometer (Invitrogen). One microgram of RNA was reverse transcribed using an iScript cDNA synthesis kit (Bio-Rad Laboratories).

### Quantitative real-time PCR.

Relative gene expression levels were determined using CFX96 real-time PCR (RT-PCR; Bio-Rad). Expression of *reg3β* and *reg3γ* was determined relative to that of the housekeeping gene *β-actin*. The quantitative RT-PCR (qRT-PCR) was performed with SYBR green PCR master mix (Bio-Rad). Primers for amplification of *reg3β* (oVPL1313 and oVPL1314), *reg3γ* (oVPL1315 and oVPL1316), and *β-actin* (oVPL1325 and oVPL1326) are listed in Table S2. Relative gene expression of the *reg3* gene in the jejunum tissues compared to *β-actin* was determined by the Relative Expression Software Tool (REST), which allows comparison of gene expression between groups of animals (26).

### Statistical analysis.

Analysis of variance (ANOVA) was used for data analysis, with Tukey’s honestly significant difference (HSD) *post hoc* comparison to investigate differences in body weight, percent fat pad and liver weight, liver triglyceride, and plasma IL-22. Significance in comparisons between groups was analyzed by *t* test. A significant difference was considered when the *P* value was lower than 0.05.
