# Supplementary material for: Secretion of Recombinant Interleukin-22 by Engineered Lactobacillus reuteri Reduces Fatty Liver Disease in a Mouse Model of Diet-Induced Obesity
Source: mSphere. 2020 Jun 24;5(3):e00183-20. doi: 10.1128/mSphere.00183-20 (PMC7316485; doi:10.1128/mSphere.00183-20)
Supplement: TABLE S1 [file mSphere.00183-20-st001.docx]

| **Strains** | **Characteristics** | **Source** |
| --- | --- | --- |
| *E. coli* EC1000 | In trans RepA provider; Kan^R^ | Jan Kok |
| *L. reuteri* VPL1014 | *L. reuteri* ATCC PTA 6475 wild-type, human breast milk isolate | BioGaia AB |
| *L. reuteri* VPL3461 | *L. reuteri* 6475 harbouring pVPL3461 for mIL-22 expression | This study |
| *L. reuteri* VPL3778 | *L. reuteri* 6475 harbouring pVPL3776 | This study |
| *L. reuteri* VPL31125 | *L. reuteri* 6475 harbouring pVPL31125 | This study |
| **plasmids** | **Characteristics** | **Source** |
| pJP028 | Em^R^, derivative of pNZ8048 containing promoter from *L. reuteri* SD2112, signal peptide from *L. reuteri* JCM1112, and LPXTG (cell wall anchor domain) | Rob Britton |
| pVPL3461 | Em^R^, derivative of pJP028 omitting LPXTG domain and harbouring *mIL-22* gene expression cassette | This study |
| pVPL3776 | pJP028:SP (signal peptide sequence):*mIL-22*:3X FLAG | This study |
| pVPL31125 | pJP028:*mIL-22*:3X FLAG | This study |
