# Supplementary figures and images for: Secretion of Recombinant Interleukin-22 by Engineered Lactobacillus reuteri Reduces Fatty Liver Disease in a Mouse Model of Diet-Induced Obesity
Source: mSphere. 2020 Jun 24;5(3):e00183-20. doi: 10.1128/mSphere.00183-20 (PMC7316485; doi:10.1128/mSphere.00183-20)

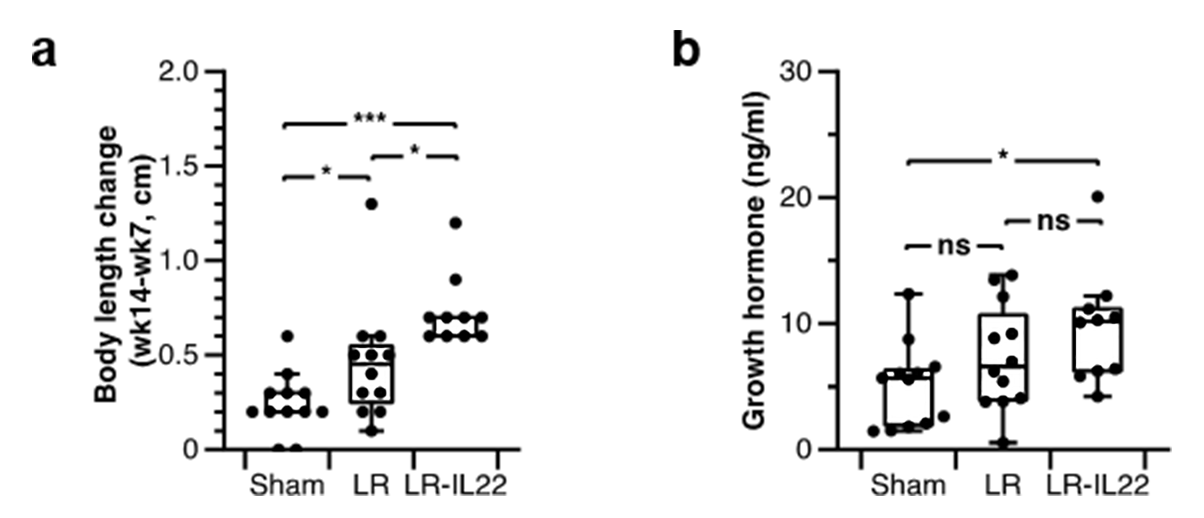

Supplement: FIG S1 [file mSphere.00183-20-sf001.tif]
