# Supplementary material for: Secretion of Recombinant Interleukin-22 by Engineered Lactobacillus reuteri Reduces Fatty Liver Disease in a Mouse Model of Diet-Induced Obesity
Source: mSphere. 2020 Jun 24;5(3):e00183-20. doi: 10.1128/mSphere.00183-20 (PMC7316485; doi:10.1128/mSphere.00183-20)
Supplement: TABLE S2 [file mSphere.00183-20-st002.docx]

| **Oligonucleotides** | **DNA sequence** |
| --- | --- |
| oVPL329 | attccttggacttcatttactgggtttaac |
| oVPL363 | taatatgagataatgccgactgtac |
| oVPL1201 | gattattcttcgataacatgggtttcactctccttctaca |
| oVPL1219 | ttcatggggatgaatgcttctgctaatacattaccagttaatactcgttg |
| oVPL1220 | cttggttttctaattttggttcaaagatcaaacacaagcattacgtaaactc |
| oVPL1221 | gcttgaaacgttcaattgaaatggca |
| oVPL1222 | tgtaaaaccaataaggactgaagc |
| oVPL1313 | actccctgaagaatataccctcc |
| oVPL1314 | cgctattgagcacagatacgag |
| oVPL1315 | atgcttccccgtataaccatca |
| oVPL1316 | ggccatatctgcatcataccag |
| oVPL1325 | ggctgtattcccctccatcg |
| oVPL1326 | ccagttggtaacaatgccatgt |
| oVPL2113 | cacgatattgattataaagatgatgatgataaatgatctttgaaccaaaattag |
| oVPL2114 | atctttataatcaccatcgtgatctttataatcaacacaagcattacgtaaactca |
| gVPL1 (*mIL-22*) | ATTCATGGGGATGAATGCTTCTGCTAATACATTACCAGTTAATACTCGTTGTAAATTAGAAGTTAGTAATTTTCAACAACCATATATTGTTAATCGTACTTTTATGTTAGCTAAAGAAGCTAGTTTAGCTGATAATAATACTGATGTTCGTTTAATTGGTGAAAAATTATTTCGTGGTGTTAGTGCTAAAGATCAATGTTATTTAATGAAACAAGTTTTAAATTTTACTTTAGAAGATGTTTTATTACCACAAAGTGATCGTTTTCAACCATATATGCAAGAAGTTGTTCCATTTTTAACTAAATTAAGTAATCAATTAAGTAGTTGTCATATTAGTGGTGATGATCAAAATATTCAAAAAAATGTTCGTCGTTTAAAAGAAACTGTTAAAAAATTAGGTGAAAGTGGTGAAATTAAAGCTATTGGTGAATTAGATTTATTATTTATGAGTTTACGTAATGCTTGTGTTTGATCTTTGAACCAAAATTAGAAAACCAAGG |
| Signal peptide (SP) | ATGTTATCGAAGAATAATCGAAAGGAACAATTCCGGAAACAAGAGCCGAAAAAGCAACGTTTTGCAATTAAAAAGCTCACTGTCGGAGTTGCTTCAGTCCTTATTGGTTTTACATTCATGGGGATGAATGCTTCTGCTAATACA |
| 3X FLAG | GATTATAAAGATCACGATGGTGATTATAAAGATCACGATATTGATTATAAAGATGATGATGATAAA |
